# Anthropometric, physical activity, and psychological characteristics of Korean adults with and without developmental coordination disorder (DCD)

**DOI:** 10.3389/fnhum.2023.1280356

**Published:** 2023-12-21

**Authors:** Min Joo Kim, Soo Mi Nam, Byeol Kim, Ilhyeok Park, Jaebum Park, Jae Kun Shim

**Affiliations:** ^1^Department of Mechanical Engineering, Kyung Hee University, Yongin-si, Republic of Korea; ^2^Division of Sports Science, Hanyang University, Ansan-si, Republic of Korea; ^3^Institute of Sport Science, Seoul National University, Seoul, Republic of Korea; ^4^Department of Physical Education, Seoul National University, Seoul, Republic of Korea; ^5^Department of Physical Education, and Advanced Institute of Convergence Science, Institute of Sport Science, Seoul National University, Seoul, Republic of Korea; ^6^Department of Kinesiology, and Neuroscience and Cognitive Science Program, The University of Maryland, College Park, MD, United States

**Keywords:** developmental coordination disorder, adults, dyspraxia, physical activity, GPAQ, psychological characteristics

## Abstract

Developmental Coordination Disorder (DCD), also known as Dyspraxia, is characterized by movement difficulties in individuals without discernible neurological disorders or identifiable medical conditions. Previous studies from various countries have highlighted disparities in anthropometric, physical activity, and psychological characteristics between children diagnosed with DCD and their typically developing (TD) peers. These differences are influenced by sociocultural norms and geographical locations. However, little attention has been given to scrutinizing analogous differences in adult populations, particularly within Republic of Korea. This study aims to address this knowledge gap by employing a battery of questionnaires to assess anthropometric, physical activity, and psychological traits in a cohort of 377 Korean adults, encompassing those with DCD (*n* = 54) alongside TD counterparts (*n* = 323). It was hypothesized that Korean adults with DCD would exhibit higher body mass index and lower ratings in physical activity and psychological characteristics than TD, consistent with the previous studies performed in other countries on children. The results showed no statistically significant differences between the DCD and TD groups in anthropometric characteristics such as weight (kg), height (cm), and body mass index. The prevalence of walking and biking for daily commuting in daily routines within Korean society might have contributed to the mitigation of anthropometric among individuals with/without DCD. Statistically significant differences were found in physical activity levels at work and recreational settings, as shown in physical activity scores and duration. The DCD group also displayed lower scores across several psychological characteristics, including exercise adherence, intrinsic motivation, self-efficacy, physical self-concept, exercise expectations, and intrinsic regulation. These findings underscore the necessity of incorporating sociocultural dynamics when investigating anthropometric, physical activity, and psychological characteristics in adults with DCD. Their perceived difficulties in fine motor skills were also significantly poor than TD. Future research studies are warranted to elucidate the underlying mechanisms driving the observed patterns in this study, thus contributing to a more nuanced comprehension of how DCD manifests within specific sociocultural contexts.

## 1 Introduction

Developmental Coordination Disorder (DCD) is a commonly diagnosed neurodevelopmental disorder, and the diagnostic and statistical manual of mental disorders-5 (DSM-5) is utilized for the clinical identification of DCD (American Psychiatric Association [APA], 2013). Notably, DCD affects motor skills and coordination, specifically called dyspraxia. These symptoms typically emerge in childhood, while the symptoms are partly extended to adulthood (American Psychiatric Association [APA], 2013). The prevalence of DCD has primarily been documented within the pediatric population, with a prevalence of 5–6% (Blank et al., 2019). However, prevalence rates among school-aged children exhibit significant variability influenced by factors such as country and ethnicity, spanning from 1.4 to 32.8% (Wright and Sugden, 1996; Lingam et al., 2009; Tseng et al., 2010; Valentini et al., 2015; De Milander et al., 2016; Amador-Ruiz et al., 2018). The prevalence of DCD within the adult population remains relatively underexplored. Nonetheless, some studies suggest that approximately 75% of adults who were diagnosed with DCD during childhood may continue to experience the condition into their adulthood years (Visser et al., 1998; Kirby et al., 2008). However, this prevalence might underestimate actual instances because of possible under-recognition, stemming from medical professionals needing more familiarity with the condition (Wilson et al., 2013). These differences are due to the methodology, cut-off criteria, assessment tool, and participant characteristics, as pointed out in previous studies (Valentini et al., 2015). Cultural background was a possible constraint when studying the prevalence of DCD (Tsiotra et al., 2006). Diagnosing DCD is a multidisciplinary effort involving collaboration between child psychiatrists, developmental pediatricians, child neurologists, and specialized therapists skilled in occupational or physical therapy. This approach ensures accurate diagnosis while adhering to the DSM-5 published by the American Psychiatric Association (American Psychiatric Association [APA], 2013).

Individuals contending with DCD encounter challenges in acquiring fundamental motor skills essential for holistic development. Growing research suggests that children with DCD are associated with difficulties in sensory processing and integration, which can hinder effective interaction with their surroundings (Goyen et al., 2011; Allen and Casey, 2017). Furthermore, children with DCD often exhibit diminished function in visual perception and motor planning (Goyen et al., 2011). Children with DCD manifest heightened tactile (Loh et al., 2011) and movement sensitivities, as well as tendencies toward under responsiveness and sensation seeking, compared to typically developing individuals (TD) (Zwicker et al., 2010). Consequently, their ability to perform everyday tasks, such as catching a ball or writing, is significantly compromised, impacting overall functional autonomy (Engel-Yeger and Hanna Kasis, 2010). Additionally, a prevailing pattern indicates that DCD frequently co-occurs with other disorders, including Attention-Deficit/Hyperactivity Disorder (ADHD), Autism Spectrum Disorder (ASD), and specific learning disabilities (Edwards et al., 2011; Blank et al., 2012; Zwicker et al., 2013b). Research on adults with DCD has received relatively little attention compared to children. A total of 75% of children with DCD continue to experience DCD into adulthood (Kirby et al., 2008).

Developmental Coordination Disorder exerts a profound and enduring impact, resonating across the lifespan and encompassing a broad spectrum of domains, including social interactions, physical and mental well-being, educational and professional achievements, and overall health-related quality of life (Karras et al., 2019). These far-reaching effects underscore the significance of understanding DCD, especially in the context of adulthood. Therefore, even as adults, individuals with DCD tend to avoid tasks that require motor skills due to slow and clumsy movements, resulting in lower levels of participation in routine physical activities and decreased quality of life (Cousins and Smyth, 2003; Zwicker et al., 2013a; Engel-Yeger, 2020). Furthermore, the challenges they face extend to basic motor skills essential for daily life, encompassing tasks like organizing, planning, time management, handwriting, using technological devices such as smartphones, and driving (Losse et al., 1991; de Oliveira and Wann, 2011; Kirby et al., 2011). This enduring pattern highlights the need for comprehensive understanding and effective intervention strategies. In alignment with well-established correlations between mental wellbeing and self-esteem in adolescents (Harrowell et al., 2017) and adults with DCD exhibit an elevated vulnerability to mood disorders (Sigurdsson et al., 2002; Harris et al., 2015; Verlinden et al., 2023). Moreover, individuals with DCD may display physical signs, including a higher prevalence of overweight or obesity in both children (Hendrix et al., 2014; Zhu et al., 2014; Yam et al., 2022) and adults with DCD (Wagner et al., 2011; Verlinden et al., 2023). These multifaceted challenges emphasize the complex nature of DCD and the need for holistic research and support.

In the absence of a conclusive medical intervention or remedy for DCD, about 75% of those affected persist in experiencing DCD throughout adulthood (Kirby et al., 2008; Reid, 2020). Yet, comprehension regarding the shifts in the physical, psychological, and behavioral traits among adults with DCD is minimal (Meachon et al., 2022). Considering the inherent connection between child development and cultural elements (e.g., traditions, religion, and family contexts) that shape experiences (Hedegaard, 2011), it becomes conceivable that some individuals with DCD might have unknowingly engaged in activities that inadvertently aided in improving their condition. The interplay of relationships in daily life, influenced by culture and socio-historical circumstances, shapes developmental progress (Nelson and Iwama, 2010; Hedegaard, 2011).

The belief that proficiency in fundamental motor skills correlates with enhanced socioeconomic conditions is widespread (Armstrong et al., 2011; Pienaar et al., 2015). For instance, East Asian countries, where the norm is to use chopsticks for eating, foster distinct fine motor skills encompassing active muscle control, focused concentration, and adept visual and motor coordination (Ohtoshi et al., 2008; Lee et al., 2019). Consequently, even individuals diagnosed with DCD, raised in East Asian cultural contexts, might showcase comparable or superior fine motor skills and hand-eye coordination compared to their non-East Asian TD peers (Chow et al., 2001). Another example pertains to physical inactivity, where exercise is prescribed as a treatment for children with DCD (Smits-Engelsman et al., 2021). The report from the World Health Organization (WHO) suggests that socioeconomic culture can influence each country’s physical activity levels and their trends in difference countries (World Health Organization [WHO], 2022). This reality emphasizes how the vast array of cultural contexts in various nations might pose a challenge to established observations concerning the disparities in physical, psychological, and behavioral aspects between individuals with DCD and TD, necessitating the adaptation of diagnosis criteria and mechanisms accordingly.

Nonetheless, there is a need for more investigation into how cultural factors influence physical, psychological, and behavioral traits in DCD. The distinctive cultural aspects of Republic of Korea may contribute to the development of fine motor skills in individuals with DCD. However, considering the findings from the Global Status Report on Physical Activity 2022, which reported relatively high physical activity rates of 70% for adult males and 59% for adult females in Republic of Korea, it is noteworthy that a vast majority of Korean adolescents aged between 11 and 17 years (91% male and 97% female) are classified as physically inactive according to the World Health Organization’s 2023 report. This suggests the potential presence of a significant motor skills gap between Korean individuals with DCD and their typically developing counterparts. When comparing Korea’s rates of physical inactivity with those of neighboring and other developed countries, a distinct trend emerges. Republic of Korea exhibits markedly higher levels of physical inactivity compared to other East Asian nations. Specifically, among adolescents, China and Mongolia report male-to-female inactivity rates ranging from 80 to 89% and 74 to 83%, respectively. For adults, China and Mongolia register male-to-female inactivity rates spanning from 12 to 16% and 18 to 19%, respectively. In contrast, Korea’s adult population demonstrates levels of inactivity more closely aligned with those found in developed Western countries like the United States (32–48%) and the United Kingdom (32–40%). However, these Western counterparts report significantly lower male-to-female inactivity rates among adolescents, ranging from 64 to 81% in the United States and from 75 to 85% in the United Kingdom (World Health Organization [WHO], 2023, Country Profile). These rates underscore the complexity of assessing fine motor skills and physical activity related to DCD on a global or regional scale. To enhance the systematic diagnosis of the DCD population, it is imperative to conduct country-specific investigations that account for sociocultural factors.

Considering these distinct Korean socioeconomic conditions, cultural attributes, and physical activity patterns, our research seeks to assess and compare the anthropometric, physical activity, and psychological characteristics of adults with DCD and TD peers. It was hypothesized that Korean adults with DCD would exhibit higher body mass index and lower ratings in physical activity and psychological characteristics than TD, consistent with the previous studies performed in other countries on children.

## 2 Materials and methods

### 2.1 Participants

This research study was approved by the Institutional Review Board at Kyung Hee University (IRB No. KHGIRB-21-342). A total of 540 university students, aged between 18 and 24 years, were recruited using email outreach, flyer distribution, online postings on university student community platforms, and cross-institutional word-of-mouth referrals. All participants provided informed written consent. After undergoing a comprehensive three-stage screening process guided by the exclusion criteria outlined in Section “2.3 Procedures,” the final cohort consisted of 377 participants.

### 2.2 Assessment tools

Three distinct sets of questionnaires were administered with the specific objectives of (1) categorizing participants into DCD and TD groups, (2) assessing physical activity patterns, and (3) evaluating perceived psychological characteristics. The administration of all questionnaires was facilitated through Google Forms. This section includes brief descriptions of each questionnaire set.

#### 2.2.1 Participant group classification

The Adult Developmental Co-ordination Disorder/Dyspraxia Checklist (ADC) designed to screen adults for DCD (Kirby et al., 2010) was administered to classify participants into DCD and TD groups. ADC consisted of three subparts: part A for as a child, part B for current symptoms, and part C for current symptoms manifested by others. Participants indicated the frequency of these difficulties by marking on a Likert scale with options “never” [1], “sometimes” [2], “often” [3], or “always” [4]. Scores for each scale were then summarized, where lower scores indicate better performance. The cut-off was set at 80 points. The ADC study conducted by Kirby et al. (2010) reported strong internal reliability for each of the three subparts: part A (α = 0.91), part B (α = 0.87), and part C (α = 0.90) (Kirby et al., 2010). Their study also established the tool’s validity by demonstrating significant correlations between ADC’s subparts and the Handwriting Proficiency Screening Questionnaire (HPSQ; Rosenblum, 2008) (subpart A: *r* = 0.68; subpart B: *r* = 0.75; subpart C: *r* = 0.71; *p* < 0.001) (Kirby et al., 2010). In order to ensure the applicability of the ADC in the Korean context, a collaborative effort was undertaken with the original authors of the ADC study and bilingual professors who were not connected to this study. This collaborative effort focused on the translation and adaptation of the questionnaire, taking into consideration the specific characteristics of the Korean population. To ensure the applicability of ADC in the Korean context, a collaborative effort was undertaken with the original authors, orchestrating the translation and adaptation of the questionnaire while staying attuned to the specific nuances of the Korean population. The questionnaire was translated through a collaborative effort involving our research team and external experts, following a structured three-stage process. The initial translation underwent stringent scrutiny under the evaluation of two bilingual Korean professors specializing in physical education and kinesiology and are currently based in the United States. Building on their valuable insights, a panel of four experts conducted a second revision. A third iteration of revision and review concluded the translation process, ultimately leading to the development of the Korean version of the ADC (Kim et al., 2023). Reliability and validity of the Korean version of the ADC have not been conducted.

The DSM-5 outlines four essential criteria for defining DCD, which encompass the presence of a motor coordination skills deficit (criterion A), difficulties in motor skills exhibited in daily activities and school settings (criterion B), the onset of symptoms during the early developmental period (criterion C), and the exclusion of other medical conditions or diagnoses (criterion D). To assess participants’ motor skill development, we employed the ADC questionnaire, which is recognized for its ability to provide insights into criteria A, B, and C of the DSM-5 (Meachon et al., 2022). Additionally, we addressed criterion D, which relates to excluding DCD that could be better explained by another medical cause, by excluding ADC questionnaire responses from participants with other medical diagnoses.

#### 2.2.2 Body mass index (BMI)

Body mass index (kg/m^2^) was computed based on participants’ self-reported weight (in kilograms) and height (in centimeters), taking into account the constraints posed by the pandemic.

#### 2.2.3 Assessment of physical activities

The assessment of physical activity characteristics was conducted using the Global Physical Activity Questionnaire (GPAQ), a tool developed by the World Health Organization to comprehensively analyze distinct physical activity patterns and engagement levels (Armstrong and Bull, 2006). The GPAQ is a widely used assessment tool for measuring physical activity levels (Cleland et al., 2014). The GPAQ comprises 16 questions spanning the domains of (1) occupational activity, (2) recreational activity, and (3) travel to and from places. These domains are further broken down into six sub-domains, including (1) vigorous work [e.g., lifting or carrying heavy objects (approximately 20 kg or more)], (2) moderate work [e.g., repetitive lifting and moving of light objects (less than approximately 20 kg)], (3) vigorous recreation [e.g., running], (4) moderate recreation [e.g., fast walking], (5) transport [e.g., riding a bike], and (6) sitting [e.g., sitting at a desk]. We employed the Korean version of GPAQ (Lee et al., 2020). The validity of the Korean GPAQ demonstrated a significant correlation with accelerometer data (*r* = 0.34, *p* < 0.01), and its reliability exhibited moderate agreement for each domain, with Cohens’ kappa values ranging from 0.38 to 0.70 (Lee et al., 2020). Participants were asked to specify their weekly engagement frequency in each category, providing details on the average duration in hours and minutes, along with the intensity (moderate or vigorous) of the activity. Vigorous activity entailed high-intensity physical activities that substantially elevated heart rate or caused heavy breathing. In contrast, moderate activity refers to moderately intense physical activities, leading to a mild increase in heart rate or slightly heavier breathing. The intensity and duration of physical activity within each domain were used to calculate the overall physical activity volume (metabolic equivalent of task-minute per week, [MET-min/week]) using the formula provided (World Health Organization [WHO], 2012). The tool demonstrates acceptable reliability and validity, incorporating adaptations to suit diverse populations across various countries and cultures (Bull et al., 2009; Trinh et al., 2009; Herrmann et al., 2013), inclusive of the specific context of Republic of Korea (Lee et al., 2020).

#### 2.2.4 Assessment of psychological characteristics

A total of eight questionnaires were employed to assess participants’ psychological characteristics across domains such as exercise adherence, intrinsic motivation, self-efficacy, physical self-concept, and intrinsic regulation, as summarized in Table 1. All questionnaires were completed using a Likert scale, and the scores were calculated as the average of the responses.

**TABLE 1:** The list of questionnaires used to measure psychological characteristics.

Measurement category	Questionnaire	Number of questions	Reference scale
Exercise adherence	Physical activity scale (PAS)	4	5
	Exercise adherence questionnaire (EAQ)	15	3
	Achievement goal questionnaire-physical education (AGQ-PE)	4	7
Intrinsic motivation	Intrinsic motivation inventory (IMI)	5	7
Self-efficacy	Motivated strategies for learning questionnaire (MSLQ)	6	7
Physical self-concept	Physical self-description questionnaire (PSDQ)	40	6
Exercise expectations	Outcome expectations for exercise scale (OEE)	9	5
Intrinsic regulation	Behavioral regulation in exercise questionnaire-3 (BREQ-3)	4	5

Exercise adherence pertains to consistent engagement in exercise activities and is often evaluated based on exercise frequency, intensity, and duration (Dishman, 1994). In this study, exercise adherence was gauged through the utilization of the Physical Activity Scale (PAS), Exercise Adherence Questionnaire (EAQ), and the Achievement Goal Questionnaire-Physical Education (AGQ-PE). The PAS was invented to assess their perceived exercise affecting physical activity adherence (Armitage and Sprigg, 2010), and the Korean version of the PAS comprising four questions with a 5-point Likert scale was modified and validated for the Korean university students (Park and Yoo, 2014). Its validity (*r* > 0.76) and reliability (α = 0.80) of the Korean version were reported (Park and Yoo, 2014). In this study, we affirmed these findings with our calculated validity (KMO = 0.83) and reliability (α = 0.96). The EAQ, introduced in 1991 to assess predisposing, enabling, and reinforcing factors affecting physical activity adherence, was validated for the Korean population (Oh et al., 2000). Its validity (KMO > 0.75) was high, and reliability (α > 0.63) was significant (Oh et al., 2000). In this study, validity (KMO = 0.91) and reliability (α = 0.90) were calculated. Comprising 15 questions, the EAQ employs a 3-point Likert scale for responses. The total score is computed by summing all the assigned points (Corbin and Lindsey, 1994). The AGQ-PE encompasses 31 items and utilizes a 7-point Likert scale, ranging from 1 (not at all true for me) to 7 (very true for me). The questionnaire covers eight psychological traits: performance-approach goals, mastery-approach goals, performance-avoidance goals, mastery-avoidance goals, social responsibility goals, social relationship goals, and effort and persistence. Our choice of this questionnaire is underpinned by its established validity and reliability within the Korean context (Park and Lee, 2010), allowing us to glean insights into individuals’ exercise adherence attitudes amid the challenges posed by physical activities. As a result, our focus centered on the persistence domain extracted from the original questionnaire, and we have included the pertinent reference (Guan et al., 2006; Park and Lee, 2010). Its validity (KMO = 0.82) and reliability (α = 0.88) of the Korean version were reported (Park and Lee, 2010). Our assessment reported KMO = 0.81 for validity and α = 0.88 for reliability.

The Intrinsic Motivation Inventory (IMI) is a comprehensive measurement tool designed to evaluate an individual’s experience with a specific activity. It has been utilized in numerous experiments exploring intrinsic motivation and self-regulation (Ryan, 1982; Plant and Ryan, 1985; McAuley et al., 1989). The IMI encompasses four distinct subscale categories: interest/enjoyment, perceived competence, perceived choice, and pressure/tension. Considering that the interest/enjoyment subscale is recognized as a self-report measure of intrinsic motivation (McAuley et al., 1989), our study specifically employed the interest/enjoyment subscale and adapted the questions to pertain to exercise. The IMI employs a 7-point Likert scale, where higher scores indicate stronger agreement with the posed questions. The validity (factor loading >0.45) and reliability (α > 0.74) of the Korean version were reported (Um and Kim, 2003). In this study, validity (KMO = 0.94) and reliability (α = 0.94) were calculated.

For the assessment of self-efficacy, which signifies an individual’s confidence in their ability to perform a task, the Motivated Strategies for Learning Questionnaire (MSLQ) was employed. The MSLQ is a self-report tool crafted to evaluate college students’ motivational orientations and their utilization of diverse learning strategies within university courses. Comprising two distinct sections, the MSLQ covers both motivation and learning strategies. The motivation section delves into students’ objectives and the value they attribute to a particular course, their perceptions regarding their competence to excel in the course, as well as their level of test-related anxiety. Within this questionnaire, higher scores correlate with increased self-efficacy levels (Pintrich et al., 1991). Our study utilized the Korean version of MSLQ, which had been modified and validated for the Korean population (Jung and Park, 2013). The validity (GFI = 0.90) of the Korean version was reported (Park and Lee, 2012). In this study, validity (KMO = 0.91) and reliability (α = 0.96) were calculated.

The Physical Self-Description Questionnaire (PSDQ) is a multidimensional instrument crafted to assess physical self-concept across 11 distinct scales: strength, body fat, activity, endurance/fitness, sports competence, coordination, health, appearance, flexibility, global physical self-concept, and global esteem, comprising a total of 70 items (Marsh, 1996). Our study employed a Korean version of the PSDQ, which entails 40 items instead of the original 70 items (Kim, 2001). Its validity (factor loading >0.53) and reliability (*r* > 0.74) of the Korean version were reported (Kim, 2001). This study calculated validity (KMO = 0.92) and reliability (α = 0.95).

The Outcome Expectations for Exercise Scales (OEE) draw from Bandura’s self-efficacy theory, assessing an individual’s convictions regarding the anticipated results of engaging in a particular behavior. This measurement is founded on the premise that outcome expectations significantly impact exercise behavior among adults (Resnick et al., 2000). Comprising a 9-item 5-point Likert scale, the OEE scale encompasses ratings from 1, representing low outcome expectations for exercise, to 5, signifying strong outcome expectations for exercise and physical activity. All items of the scale were employed in our study. The Korean version of OEE, which has been reported to have validity (factor loading >0.53) and reliability (*r* > 0.74) (Kim, 2001), was employed in this study. For our research, we conducted validity (KMO = 0.89) and reliability (α = 0.87) calculations.

The Behavioral Regulation in Exercise Questionnaire-3 (BREQ-3) is a widely recognized assessment tool that encompasses a range of exercise motivation types, which include motivation, external regulation, introjected regulation, identified regulation, integrated regulation, and intrinsic motivation (Markland and Tobin, 2004; Wilson et al., 2007). In our investigation, our focus was on evaluating intrinsic regulation. Therefore, we selected a specific subset of questions that pertain to intrinsic motivation, which signifies engagement in an activity driven by its inherent enjoyment and satisfaction. This subset comprises four items. The Korean version of this particular subset has previously established its validity (factor loading >0.68) and reliability (*r* > 0.76) (Lim and Hu, 2011). For our current study, we performed calculations for both validity (KMO = 0.86) and reliability (α = 0.96).

### 2.3 Procedures

After receiving informed consent from each participant, individuals in the DCD group scored 80 points or higher on the ADC. Participants also completed online surveys to provide their self-reported physical activity levels and psychological characteristics, elucidated in section “2.2 Assessment tools”.

**FIGURE 1:**
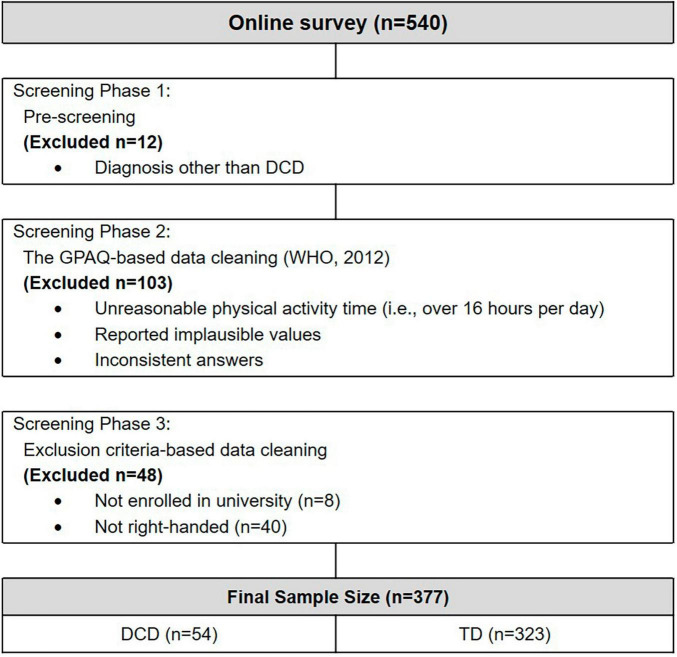
Flow chart for the exclusion process.

Our research team employed a 3-stage screening procedure. The study was conducted using an online survey methodology, initially collecting responses from 540 participants. The first phase of our data screening involved pre-screening to eliminate individuals with diagnoses other than DCD from ADC questionnaire. A total of 12 participants were excluded from the study (Figure 1). This exclusion comprised 10 participants with ADHD, 1 with dyspraxia, and 1 with dysthymic disorder. A recent international recommendation (Blank et al., 2019) states that DCD and dyspraxia are not the same, so we excluded individuals with other diagnoses. The subsequent screening phase encompassed the GPAQ data cleaning following its manual (World Health Organization [WHO], 2012). This process identified instances of unreasonable physical activity time (e.g., exceeding 16 h per day), implausible values, and conflicting responses, leading to the removal of 103 participants. These exclusions adhered to predefined criteria aimed at ensuring the accuracy and reliability of the physical activity data. In the last phase of the exclusion process, only those currently enrolled at a university and were right-handed were retained for the study. This criterion was informed by research on handedness, which revealed that left-handed individuals may face difficulties in their everyday experiences (Thomas et al., 2019). This methodological approach was undertaken to facilitate a nuanced comparative analysis amongst subjects with similar psychological maturation and socioeconomic standing levels. The participants who cleared all three stages of the screening procedure underwent an assessment using the ADC to classify them into either the DCD or TD group.

### 2.4 Statistics

We conducted descriptive statistics for the mean (M) and standard deviation (SD) of demographic and anthropometric features (i.e., age, height, weight, and BMI) and those variables related to ADC (i.e., total score, part A, B, and C, and subset score of fine motor skills). To test the assumptions for parametric statistics, we conducted the Shapiro-Wilk and Levene’s tests for normality and equal variance on the demographic and anthropometric features, variables related to ADC (i.e., total score, part A, B, and C, and subset score of fine motor skills), physical activity (GPAQ) and psychology (i.e., PAS, EAQ, AGQ-PE, IMI, MSLQ, PSDQ, OEE, and BREQ-3). After discovering that these assumptions were not met, we conducted a Yuen’s *t*-test between the DCD and TD groups. It’s worth noting that Yuen’s test does not rely on the assumptions of normality and equal variance. All statistical analysis was conducted in R (Version 4.3.1). Our statistical significance level was set at 0.05 for all analyses.

## 3 Results

### 3.1 Participants’ demographic and anthropometric characteristics

**FIGURE 2:**
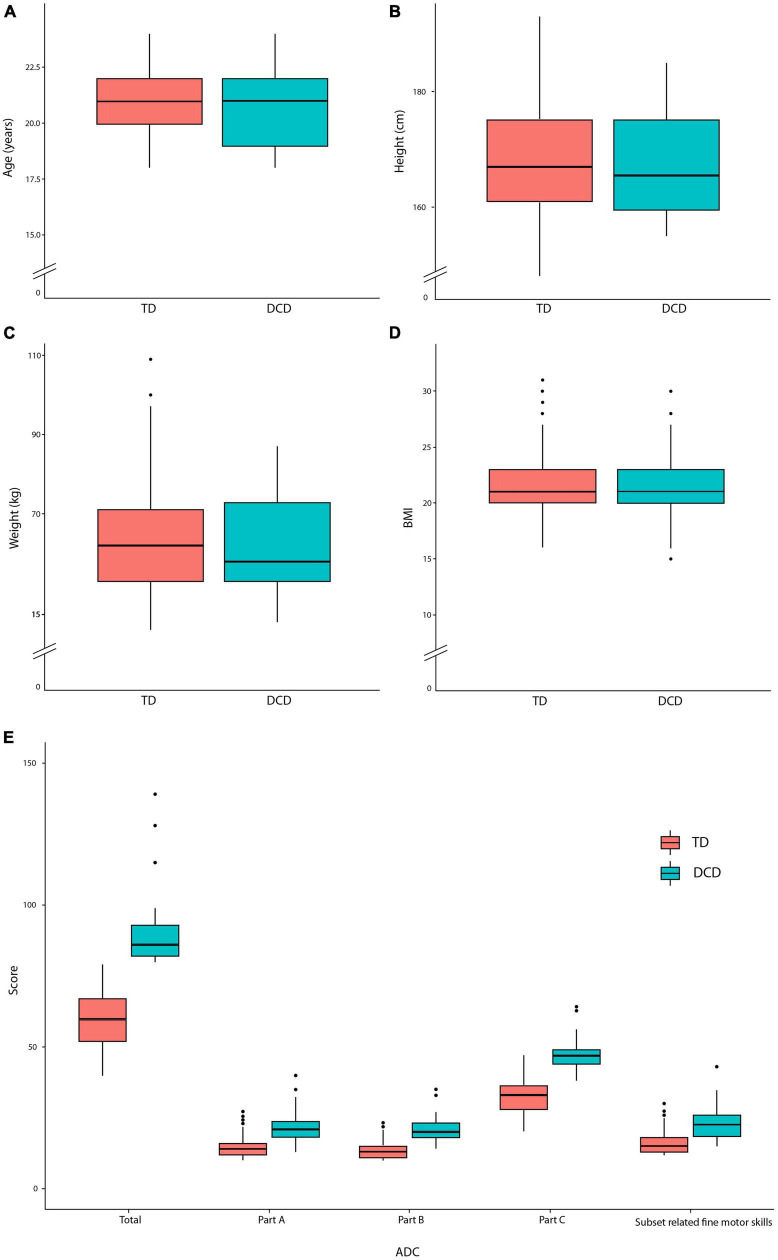
The box plots for **(A)** age, **(B)** height, **(C)** weight, **(D)** BMI and **(E)** ADC.

Among the initial 540 participants recruited for the study, 377 participants cleared all three stages of the screening procedures. This final group comprised 170 males (45.1%) and 207 females (54.9%). The average age of the participants was 20.98 years. Participants were categorized into two groups based on their total ADC scores: 54 participants with DCD (ADC score equal to or over 80) and 323 participants classified as TD (ADC score below 80) (Table 2). These numbers revealed a prevalence rate of 14% for DCD within this study. No statistically significant differences were found between the DCD and TD groups regarding age, height, weight, and BMI (Figures 2A–D). We also compared ADC scores, including their perceived fine motor skills with scores from ADC related to fine motor skills (ADC items: A-1, A-2, A-5, A-6, A-8, B-1, B-2, B-4, B-5, B-6, B-7, and C-6) (Figure 2E). We found that the DCD group showed significantly higher scores in ADC total, ADC parts A, B, and C, and subset scores related to fine motor skills (Table 2).

**TABLE 2:** The Yuen’s *t*-test results in demographic and anthropometric characteristics of DCD and TD groups.

Items		Groups [M (SD)]		*t*
		DCD	TD	
Age (years)		20.85 (1.65)	21.01 (1.67)	0.69
Height (cm)		166.92 (8.38)	167.85 (8.63)	0.80
Weight (kg)		61.87 (11.60)	62.44 (11.95)	0.01
BMI (kg/m^2^)		22.09 (3.09)	21.83 (3.24)	-0.06
ADC	Total score	89.28 (11.20)	60.04 (9.69)	-20.76***
	Part A: as a child	21.60 (5.27)	14.20 (3.34)	-12.05***
	Part B: current perception of performance	20.50 (4.29)	13.40 (2.74)	-13.06***
	Part C: current feelings	47.15 (5.30)	32.44 (5.67)	-18.38***
	Score related fine motor skills	22.15 (3.62)	15.18 (5.89)	-8.40***

ADC: The adult developmental co-ordination disorder/dyspraxia checklist.

***p < 0.001.

### 3.2 Assessment of physical activities

**FIGURE 3:**
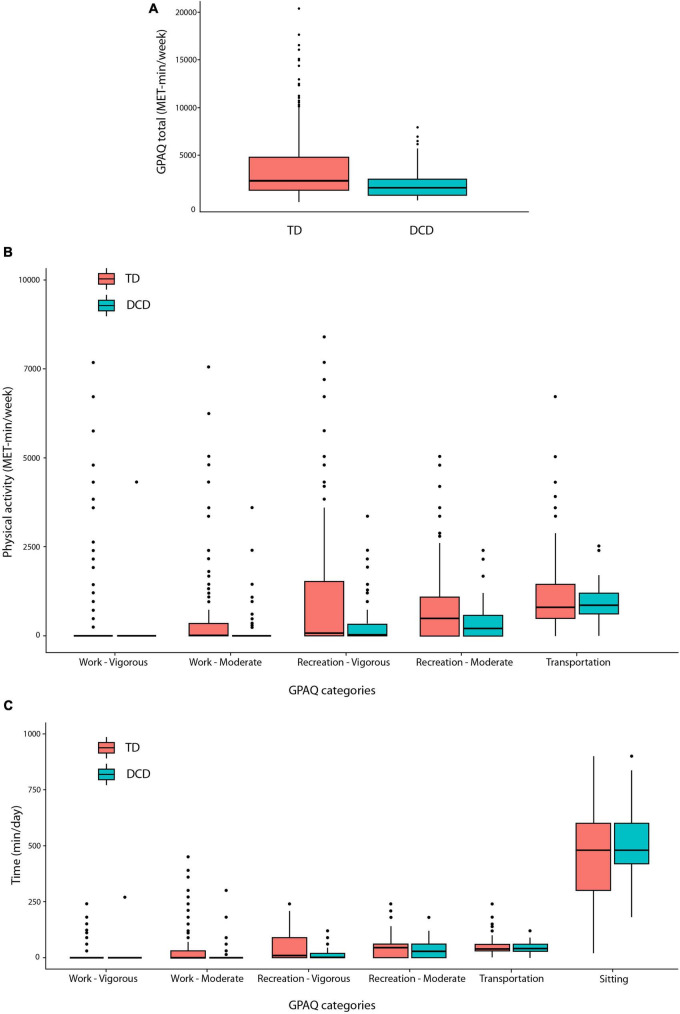
The box plots of GPAQ for **(A)** total physical activity, **(B)** physical activity, and **(C)** physical activity time per day.

We employed the GPAQ as an assessment tool for evaluating physical activities (Figure 3). The result of the GPAQ total indicated a significant difference between DCD and TD groups [*t*_(97.16)_ = 3.98; *p* < 0.001]. The DCD group exhibited statistically lower physical activity levels than the TD group. Within the DCD group, there were significantly lower levels of the work-moderate [*t*_(226.53)_ = 2.39; *p* = 0.017], recreation-vigorous [*t*_(89.27)_ = 3.53; *p* < 0.001], and recreation-moderate domains [*t*_(66.97)_ = 3.08; *p* < 0.001], except transport domain [*t*_(65.98)_ = 0.17; *p* = 0.869]. These results suggest that adults with DCD are less physically active in most domains. Further analysis of the time (minute per day) dedicated to physical activity from the GPAQ demonstrated a significant difference between the DCD and TD groups. When converted to minutes, the results also show how much less adults with DCD move compared to the TD group. Specifically, the DCD group spent significantly less time in the work-moderate [*t*_(212.27)_ = 2.03; *p* = 0.043], recreation-vigorous [*t*_(116.16)_ = 4.29; *p* < 0.001], and recreation-moderate domains [*t*_(51.78)_ = 2.32; *p* = 0.024] compared to the TD group. However, no significant differences were found in the transport [*t*_(53.98)_ = −0.14; *p* = 0.886] and the sitting [*t*_(43.06)_ = −1.87; *p* = 0.067] domains.

### 3.3 Assessment of psychological characteristics

**FIGURE 4:**
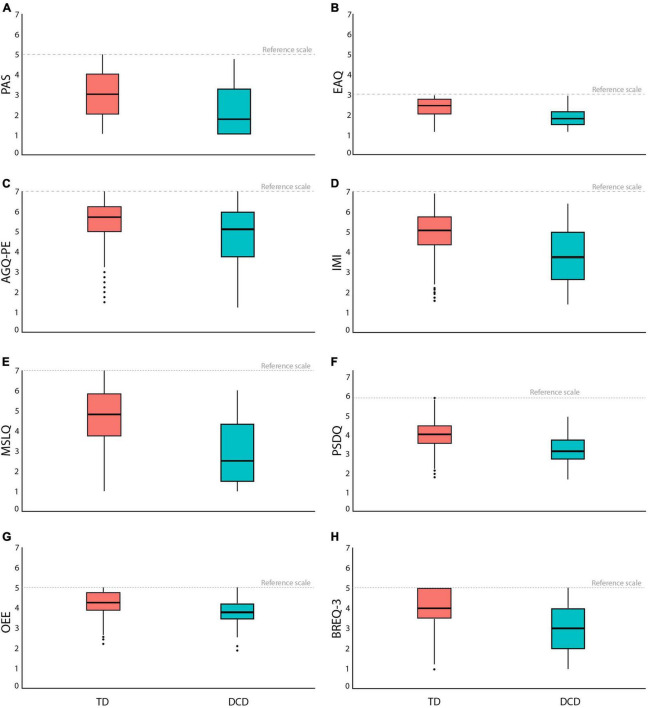
The box plots in psychological characteristics for **(A)** PAS, **(B)** EAQ, **(C)** AGQ-PE, **(D)** IMI, **(E)** MSLQ, **(F)** PSDQ, **(G)** OEE, and **(H)** BREQ-3.

We employed several questionnaires to identify psychological characteristics (Table 1). We compared across all psychological characteristics–exercise adherence, intrinsic motivation, self-efficacy, physical self-concept, exercise expectation, and Intrinsic regulation. The DCD group exhibited statistically significantly lower scores across all psychological characteristics compared to the TD group. Exercise adherence of DCD group revealed significantly lower scores than TD in PAS [*t*_(53.52)_ = 4.23; *p* < 0.001], EAQ [*t*_(41.87)_ = 6.47; *p* < 0.001], and AGQ-PE [*t*_(38.42)_ = 2.46; *p* = 0.018], suggesting that the DCD group perceived that they have lack of willpower to continue exercising and did not think they have enough support of them surroundings or family and friends to continue their exercise (Figures 4A–C). Intrinsic motivation of the DCD group showed significantly lower scores than TD [*t*_(35.07)_ = 5.00; *p* < 0.001] (Figure 4D), which suggests that the DCD group was less motivated to participate in physical activities and less confident in performing motor skills. Self-efficacy scores of the DCD group were significantly smaller than TD [*t*_(43.66)_ = 7.19; *p* < 0.001] (Figure 4E), which suggests that the DCD group showed lower self-efficacy due to poor motor skills when participating in physical activities. Physical self-concept of the DCD group presented significantly lower scores than TD [*t*_(46.41)_ = 7.29; *p* < 0.001] (Figure 4F). As we mentioned in section “3.1 Participants’ demographic and anthropometric characteristics,” the DCD group was not different from the TD group regarding physical characteristics. However, the DCD group perceived themselves as less fit and less capable of performing sports. Exercise expectation of the DCD showed significantly lower scores than the TD group [*t*_(43.97)_ = 3.62; *p* < 0.001] (Figure 4G). Although not as much as the TD group, the DCD group generally expected exercise to promote their health and benefit from exercise. Intrinsic regulation of the DCD group revealed significantly lower scores than the TD [*t*_(39.34)_ = 4.93; *p* < 0.001] (Figure 4H), suggesting that the DCD group enjoyed less participating in or doing exercise.

## 4 Discussion

In this study, we found that despite exhibiting lower levels of physical activity and having lower levels of several psychological factors related to exercise (e.g., exercise adherence, intrinsic motivation, self-efficacy, physical self-concept, exercise expectancy, and Intrinsic regulation), Korean adults with and without DCD did not differ significantly in terms of physical characteristics like height, weight, or BMI. These findings align with previous research on adults with DCD, which showed reduced physical activity (Wilson et al., 2017) and low levels of psychological wellbeing (Tal-Saban et al., 2012; Purcell et al., 2015). However, the adults with DCD in our study who exhibited lower physical activity levels maintained BMIs within the normal range, similar to the typically developing population. This contrasts with earlier studies conducted in Canada (Cantell and Crawford, 2008) and Belgium (Verlinden et al., 2023), as well as with research on adolescents with DCD in Germany (Wagner et al., 2011), which reported higher BMIs among individuals with DCD.

The participants with DCD in this study were also found to have difficulty performing motor skills from childhood. Despite the noteworthy persistence of childhood DCD into adulthood, the existing body of research on DCD has predominantly focused on the pediatric population, with estimates ranging from 2% to 30% (Lingam et al., 2009; Blank et al., 2019; Kita et al., 2020). The limited adult DCD research available mirrors many traits commonly associated with childhood DCD, including heightened frustration, diminished competency, lower self-esteem, restricted engagement in daily activities (Jarus et al., 2011; Tal-Saban et al., 2014), and a compromised quality of life (Engel-Yeger, 2020), culminating in an overwhelming emotional burden (Cairney and Veldhuizen, 2013). This congruence between childhood and adult DCD traits might have influenced the adoption of children’s DCD diagnostic tools to adults. However, the broader context of human development underscores the significant influence of culture, leading to potential diversities in the observed physical, psychological, and behavioral characteristics within DCD adult populations across varying countries. Therefore, it is crucial to comprehensively understand the cultural associations underpinning DCD symptoms and then apply them to adult DCD diagnostic tools and interventions.

Culture stands as a potent yet often underestimated determinant of development and functioning (Matsumoto, 2001). Its influence extends beyond social dynamics, impacting cognitive processes and even biological responses (Schwartz et al., 2020) while also extending to motor skills (Cintas, 1995; Chow et al., 2001). The dynamic interplay between culture and context creates an environment where unique experiences shape psychological processes. For instance, a notable study discovered a substantial correlation between national culture and BMI across a cohort spanning 53 countries (Masood et al., 2019). The influence of culture extends to self-esteem as well. Research involving teenagers and young adults from 19 to 20 countries reveals that their self-esteem is not solely based on personal values but is shaped by the alignment with value priorities prevalent in their cultural surroundings (Becker et al., 2014). This cultural imprint even reverberates in the realm of physical activity levels, with cross-country disparities evident in a study encompassing 52 countries (Bann et al., 2019).

The assumption that elevated BMI in DCD has been differed by sample and other cultural factors. Like the present study, a longitudinal examination of the DCD population in Finland did not uncover statistically significant BMI differences compared to typically developing individuals (Tan et al., 2022). Conversely, previous literature shows that adults with DCD have a higher BMI (Cantell and Crawford, 2008; Verlinden et al., 2023), so it is important to consider the participant characteristics of these studies in interpreting the conflicting results. There is a possibility that other characteristics observed in individuals with DCD population, including enduring challenges in fine and gross motor skills (Cantell et al., 2003), writing (Barnett et al., 2011), time estimation (Tal Saban et al., 2014), learning to perform new tasks (de Oliveira and Wann, 2011), academic achievements/performance (Dewey et al., 2002; Alloway, 2007; Kirby et al., 2013; Harrowell et al., 2018), and mood disorder/anxiety (Cairney et al., 2010; Purcell et al., 2015; Omer et al., 2019), may not hold true across different countries. This observation holds substantial importance in terms of its potential impact on the accuracy of DCD diagnostic tests and the strategies for interventions. Considering that diverse cultural influences might lead to distinct patterns, the necessity of potentially modifying testing methods warrants careful consideration.

Although many studies have reported a higher prevalence of obesity among DCD population (Cantell and Crawford, 2008; Ferguson et al., 2015; Kumpulainen et al., 2016), our study did not reveal any significant statistical differences in height, weight, and BMI between adults with DCD and TD groups. The previous literature related to the population with DCD and their BMI and motor skills showed individuals with DCD with higher BMI and lower motor skills (Cantell and Crawford, 2008; Chivers et al., 2013). This divergence in findings could potentially be attributed to an enduring cultural trend. A recent longitudinal study scrutinized the national obesity percentages across all genders and age groups in various regions of Republic of Korea between 2009 and 2018 (Nam et al., 2020). This investigation unveiled an escalating prevalence of obesity in both young men and women, particularly in the age range of 20–39 years (Nam et al., 2020). A plausible cultural explanation for this trend can be inferred from the statistics furnished by the Global Status Report on Physical Activity 2022: country profiles (World Health Organization [WHO], 2023), where physical inactivity in Republic of Korea was reported at 91% for male adolescents and 97% for females aged 11–17 years. Although these rates exhibit a slight reduction among adults aged 18 years and above, they remain notably high at 30% among males and 41% among females. Despite the characterization of Korean adults as physically inactive, BMI was in the normal range in all groups, and the results of this study may support the need to consider specificities such as race and culture. While our study did not identify discernible differences between the DCD and TD groups in terms of bicycle or walking use for commuting, notable contrasts emerged in occupational and leisure activity choices. The Korean adults with DCD appeared to gravitate toward pursuits that involve lower physical demands, as evidenced by fewer hours and reduced frequency of engagement. We hypothesize that when individuals find themselves in situations with limited options, the Korean DCD group tends to conform to prevailing norms. However, when presented with the choice to be less physically active, they readily opt for such alternatives. This result may be rooted in the context of our study participants, who were students living in an urban environment where walking and biking are customary modes of transportation, akin to many European countries. Consequently, they had to rely on relatively physically demanding modes of transportation. On the other hand, work is a realm where individuals can exercise independent decision-making. People can easily and independently identify jobs that entail less physical intensity and choose to avoid physically demanding roles. Given that all our participants were currently enrolled in school, it is plausible that their inclination toward occupations requiring greater physical exertion may have been limited, irrespective of their physical capabilities, motor competence, and confidence in engaging in physical activities. This aligns with the findings from our GPAQ analysis. Based on these observations, we recommend that adults with DCD diagnosis questionnaire consider emphasizing an individual’s autonomy in actions. This would involve focusing on identifying actions driven by personal choice rather than actions influenced by cultural adaptation.

Psychological variables (Table 1) exhibited significantly lower values in the group of adults with DCD, consistent with findings from previous DCD studies conducted in various countries (Sigurdsson et al., 2002; Harris et al., 2015; Verlinden et al., 2023). Our study utilized a questionnaire to assess psychological states related to participation and persistence in exercise and physical activity. The results indicated a pronounced reluctance to engage in and sustain exercise, mirroring observations in previous research (Jarus et al., 2011; Engel-Yeger, 2020). This observed reluctance to participate in physical activities and maintain exercise regimens can be seen as an extension of a recurring characteristic among individuals with DCD, which begins in childhood. Children with DCD often shy away from various physical activities and physical education classes due to their lower motor skill abilities. In our study, participants with DCD reported experiencing difficulties when performing daily activities and engaging in physical pursuits during their childhood years (see ADC Part A from Table 2). Additionally, their reduced motivation to participate in exercise, coupled with lower levels of self-efficacy and physical self-concept, may account for their decreased involvement in recreational physical activities compared to the TD population. Furthermore, our findings align with prior research, emphasizing that adults with DCD tend to exhibit lower overall psychological wellbeing compared to the general population.

The ADC questionnaire encompasses inquiries designed to assess difficulties in fine motor skills. These inquiries are rooted in extensive research highlighting challenges among adults with DCD regarding fine motor skills (Cantell et al., 2003), often translating to subpar handwriting performance (Barnett et al., 2011). Within the framework of the ADC questionnaire, we delved deeply into fine motor skills, subjecting questions related to challenges involving utensil uses for eating, handwriting, and grooming activities to meticulous statistical analysis. As mentioned in our Introduction, a question revolved around the influence of early exposure to chopstick usage in the Korean population on fine motor skill development. Yet, upon scrutinizing responses from both DCD and TD groups to fine motor skill questions within the ADC questionnaire, we discerned no statistically significant divergence in fine motor skills among Korean DCD and TD groups. This intriguing revelation suggests that cultures with a strong emphasis on honing fine motor skills may encounter difficulties in accurately identifying instances of DCD. This finding also hints at the possibility that our reported prevalence rate of 14% might be a conservative estimate, extending this trend to nations where chopstick use is prominent.

In conclusion, this study pioneers the investigation of anthropometric, physical activity, and psychological characteristics among Korean adults with DCD. While many of our findings align with global trends, deviations in specific aspects highlight the potential influence of culture on developmental trajectories, potentially leading to distinct patterns among adults with DCD. This underscores the value of comprehensive cross-cultural studies and subsequent adaptation of diagnostic mechanisms and intervention strategies. This study utilized perceived questionnaire responses to identify traits in adults with DCD and typically developing Korean adults. Our upcoming research phase will delve deeper into the social, physical, and neurological contributors and mechanisms underlying the differences between adults with and without DCD.

## Data availability statement

The raw data supporting the conclusions of this article will be made available by the authors, without undue reservation.

## Ethics statement

The studies involving humans were approved by the Institute of Review Board, Kyung Hee University. The studies were conducted in accordance with the local legislation and institutional requirements. The participants provided their written informed consent to participate in this study.

## Author contributions

MK: Conceptualization, Data curation, Funding acquisition, Investigation, Methodology, Project administration, Resources, Visualization, Writing – original draft, Writing – review and editing, Formal analysis, Supervision. SN: Data curation, Formal analysis, Methodology, Writing – original draft, Writing – review and editing. BK: Writing – original draft, Writing – review and editing. IP: Data curation, Methodology, Writing – review and editing. JP: Conceptualization, Formal analysis, Funding acquisition, Investigation, Methodology, Resources, Software, Supervision, Writing – original draft, Writing – review and editing. JS: Conceptualization, Data curation, Funding acquisition, Investigation, Methodology, Project administration, Resources, Software, Supervision, Writing – original draft, Writing – review and editing.
